# The Development of Compartmentation of cAMP Signaling in Cardiomyocytes: The Role of T-Tubules and Caveolae Microdomains

**DOI:** 10.3390/jcdd5020025

**Published:** 2018-05-03

**Authors:** Navneet K. Bhogal, Alveera Hasan, Julia Gorelik

**Affiliations:** Department of Cardiovascular Sciences, National Heart and Lung Institute, Imperial College London, London W12 0NN, UK; n.bhogal14@imperial.ac.uk (N.K.B.); alveera.hasan13@imperial.ac.uk (A.H.)

**Keywords:** cAMP, phosphodiesterase, FRET, atrial, ventricle, iPSC-CMs, T-tubule, caveolae, development

## Abstract

3′-5′-cyclic adenosine monophosphate (cAMP) is a signaling messenger produced in response to the stimulation of cellular receptors, and has a myriad of functional applications depending on the cell type. In the heart, cAMP is responsible for regulating the contraction rate and force; however, cAMP is also involved in multiple other functions. Compartmentation of cAMP production may explain the specificity of signaling following a stimulus. In particular, transverse tubules (T-tubules) and caveolae have been found to be critical structural components for the spatial confinement of cAMP in cardiomyocytes, as exemplified by beta-adrenergic receptor (β-ARs) signaling. Pathological alterations in cardiomyocyte microdomain architecture led to a disruption in compartmentation of the cAMP signal. In this review, we discuss the difference between atrial and ventricular cardiomyocytes in respect to microdomain organization, and the pathological changes of atrial and ventricular cAMP signaling in response to myocyte dedifferentiation. In addition, we review the role of localized phosphodiesterase (PDE) activity in constraining the cAMP signal. Finally, we discuss microdomain biogenesis and maturation of cAMP signaling with the help of induced pluripotent stem cell-derived cardiomyocytes (iPSC-CMs). Understanding these mechanisms may help to overcome the detrimental effects of pathological structural remodeling.

## 1. Introduction

The fascination about 3′-5′-cyclic adenosine monophosphate (cAMP), a second messenger molecule, is with its ability to activate multiple signalling pathways having different effects on cellular physiology in many cell types. Cardiomyocytes, in particular, have been studied in detail for the regulation of cAMP production as a result of extracellular stimulation [1,2]. Buxton and Bruton indicated a paradox whereby stimulation of a cell via various receptor pathways provided diverse cellular responses despite generating the same cAMP second messenger [3]. It was further elucidated, that these multiple spatially confined cAMP compartments may be the result of different protein kinase A (PKA) isoforms and other cAMP-sensitive downstream targets having restricted access to cAMP [4]. Studies have since been directed at understanding how these vital compartments of cAMP are maintained within the cell.

The localized synthesis of cAMP by one of 10 different adenylate cyclase (AC) isoforms is crucial in providing initial first step in signalling cascade, however, the manner in which cAMP is compartmentalized within the cell is essential to elicit a specific cellular response.

Saucerman et al. proposed several mechanisms that might be involved in cAMP compartmentalization including: local degradation, physical barriers, and cell shape [5]. There are many factors that come together to give rise to cAMP compartments. In this review, we focus on the role of cardiac microdomains and phosphodiesterases (PDEs).

## 2. β-Adrenergic Pathway

The heart responds to catecholamines with increases in rate, force of contraction, and speed of relaxation [6]. The main catecholamine-responsive receptors on the surface of cardiac myocytes are β-adrenergic receptors (β-ARs), an important class of G-protein-coupled receptors (GPCR). To generate these responses, β-ARs are coupled to G_s_ proteins, which activate AC and, thus, induce cAMP production. The cAMP binds to effector proteins including, PKA [7] (preferentiality PKA-RII in the case of β-AR [8]), exchange protein directly activated by cAMP (EPAC) [7], cyclic nucleotide gated ion channels (CNGCs) [9], and Popeye-domain-containing proteins (Popdc) [10]. PKA is the major effector protein and phosphorylates a large number of target proteins which are for example involved in excitation-contraction coupling (ECC) [6].

β-AR stimulation is however more complex. In the late 1960s, Lands et al. studied the activity of sympathomimetic amines in different tissues and concluded that there are two isoforms of β-ARs: β_1_-AR and β_2_-AR [11,12]. In the heart, β_1_-AR is the predominant isoform [12]. The important role of β_1_-AR for the heart was demonstrated through targeted disruption in mice. The Adrb1 null mutant displayed impaired cardiac performance and prenatal lethality [13]. In contrast, the β_2_-AR is apparently less important for the heart and more active in the respiratory system [12]. Both receptor types differ in their G-protein coupling, while β_1_-AR couples only to G_s_ proteins, β_2_-AR couples to both G_s_ and G_i_ proteins. Adrb2 mutants display normal resting heart rate and blood pressure, but develop hypertension in response to epinephrine infusion or to the cardiovascular stress induced by exercise [14]. Interestingly, human ventricular tissue display a higher expression level of β_1_-AR in comparison to β_2_-AR with a ratio of approximately 77:23 [15]. The predominance of β_1_-AR provides an explanation for the fact that mice lacking β_2_-AR experience alterations during exercise to their vascular tone and energy metabolism [14], but nothing more fatal. The low cardiac expression level of β_2_-AR together with its ability to either stimulate (via G_s_) or inhibit (via G_i_) cAMP synthesis suggests an involvement in the ‘fine-tuning’ of cardiac contractility. Importantly, β_2_-AR are thought to have protective effects on cardiac function [16]. In addition to differences in expression level and G-protein coupling, the functional properties of β-ARs can also be affected by their spatial localization at the plasma membrane [16].

An important downstream effector molecule of β-AR signalling is AC. Stimulation of β-AR leads to activation of ACs catalysing the conversion of ATP to cAMP. The two major isoforms of ACs expressed in the heart are AC5 and AC6. Patch clamp recordings of ventricular myocytes from Adcy5 (AC5) and Adcy6 (AC6) knock-out mice, demonstrated the divergent roles of these adenylyl cyclase isoforms [17]. An AC5-mediated increase of the calcium current was recorded from T-tubules, and was mediated by both β_1_-AR and β_2_-AR [17]. In contrast, AC6 was shown to interact with β_1_-AR alone. Interestingly, upon genetic ablation of Adcy5, it was shown that AC5 is protective against cardiac dysfunction in pressure overload induced cardiac hypertrophy [18], thus suggesting that the stimulation of AC5 could potentially be a protective therapeutic approach in heart failure. Introducing AC6 was able to restore the cAMP-generating capacity in a murine cardiomyopathy model [19,20]. The targeted deletion of Adcy6 caused a marked impairment of cAMP synthesis and sarcoplasmic reticulum calcium release [21]. The differing effects on cardiac function upon gene ablation suggests that these AC isoforms provide different functions to the heart.

## 3. Transverse Axial Tubular System

Important and highly-complex compartments in the cardiac myocyte are the transverse tubules (T-tubules). These deep, penetrative sacrolemmal invaginations are a characteristic of ventricular myocytes, and openings of these tubules can be visualized on the cellular surface in Z-groove structures [22]. These microdomains act as a framework for several ion channels and proteins essential for regulating the strength and synchronicity of each cardiac contraction [23]. These proteins and effector molecules are grouped in specific protein complexes along the plasma membrane [24].

The complexity of the transverse axial tubular (TAT) system has received more attention over the years, due to the presence of axial (also known as longitudinal) elements becoming more apparent [22,25]. The increase in axial elements raises questions of their functional importance. Studying healthy ventricular myocytes have demonstrated that small mammals have a more prominent T-tubular system compared to those from larger mammals [26,27]. Detailed studies in rabbits demonstrated that the diameter of the T-tubules is approximately two-fold wider than in mice [26]. This suggests that the abundant presence of T-tubules seen in mice is likely due to myocyte’s requirement to accommodate ECC proteins in order to achieve a proper contractile response. In addition, it is possible that wider T-tubules are able to accommodate the required ECC proteins in fewer structures, which provides an explanation for the patchy tubular system seen in larger animals. These structural differences between species are likely the result of the vast differences in heart rate that exists between small and large mammals; thus, the structure is adjusted to meet the species-specific requirements. Therefore, the regularity and structural environment of these microdomains is crucial for physiological signalling of receptors and ion channels in both atrial and ventricular myocytes [28,29].

It has been long thought that atrial myocytes either lack, or contain only a rudimentary TAT network [30,31,32,33,34]. However, more recently it became evident that atrial myocytes are more diverse. Gadeberg et al. have shown that in atrial myocytes of larger mammals including humans a TAT network is present, with cell size being a determinant for T-tubular density [30]. Interestingly, murine atrial myocytes display a predominantly axial tubular structure, and the diameter of these tubules is wider than in ventricular myocytes [31,32]. Moreover, the most organized atrial myocytes resemble ventricular myocytes with regard to the TAT network.

**Figure 1:**
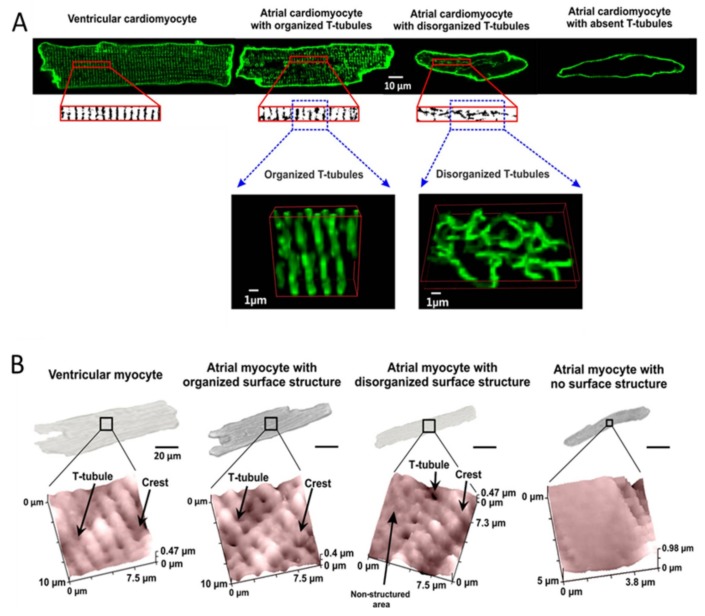
The heterogeneity of myocytes found in the rat heart. (**A**) Representative confocal images of Di-8-ANNEPS-stained myocytes from the atria and the ventricle. Atrial cells were categorized based on the T-tubule organization and density into three distinct subgroups with organized TAT, disorganized TAT and myocytes lacking all T-tubular structure. Parts of images were binarised as shown and used to calculate the density and demonstrate a clear representation of the structural changes between groups; (**B**) Topographical scans of myocytes demonstrating the occurrence of T-tubule opening at the Z-lines of myocytes in relation to their cell size. Arrows indicate T-tubules, crests, and non-structured areas. Adapted from Glukhov et al. 2015 [34].

The atria are structured as two separate “pockets”, with the right atrium containing anatomical structures (i.e., crista terminalis and sinoatrial node), which are not present in the left atrium. Yamashita et al. have shown that, although the length to width ratio remains the same, myocytes isolated from the crista terminalis are larger than from pectinate muscle [33]. Therefore, differently structured myocytes may be derived from different atrial regions. Investigations of small mammals also demonstrated the requirement of the TAT structure in the atria and further highlighted the heterogeneous structure in these myocytes [34,35]. Glukhov et al. studied rat atrial myocytes utilizing confocal microscopy with scanning ion conductance microscopy (SICM) to demonstrate the existence of three populations of myocytes: organized, disorganized, and empty cells [34] (Figure 1A). These distinct populations of atrial myocytes have been found in both small and large mammals in multiple independent studies [34,35,36].

## 4. Caveolae

Caveolae are 50–100 nM-wide flask-shaped membrane invaginations. These microdomains are dense in cholesterol and are lined with specialized scaffolding proteins, which are key features distinguishing caveolae from other lipid raft structures [32]. Caveolae are responsible for the spatial organization of signalling proteins, lipid storage, and membrane homeostasis. Although it is well known that the caveolar coat is comprised of oligomers of the scaffolding proteins caveolin and cavin, the process of assembly and recycling of these membrane domains remained debatable for many decades. Recently, Hayer et al. highlighted the multistep process of caveolae assembly [37]. This involves the homo-oligomerisation of caveolin-1 (Cav1) into 8S complexes in the Golgi where they undergo conformational changes and associate with cholesterol, thus assembling into 70S complexes. These newly-assembled caveolin scaffolds are transported to the plasma membrane where they are able to act as functional caveolar domains.

Caveolae have the ability to act as membrane reservoirs allowing the cell to increase its surface area in response to osmotic stress [38,39,40]. Furthermore, Sinha et al. and others have shown that acute mechanical stress induced by osmotic swelling or stretching, reduced the interaction of caveolin with cavin-1. It increased the concentration of free caveolin proteins at the plasma membrane, thus resulting in a rapid disappearance of caveolae [38,41]. Mohan et al. further elucidated that Cavin-1 regulates caveolae dynamics, by targeting Cavin-3 to caveolae where it interacts with the scaffolding domain of Cav1 [42]. Loss of Cavin-3 resulted in an increase in the long-term stability of caveolae at the plasma membrane, suggesting that it regulates the lifetime of caveolae.

Calaghan et al. studied the dynamic nature of caveolae in response to mechanical stimuli; subjecting adult rat hearts to left ventricular ballooning and the time-dependent effect of stretch on the distribution of caveolin-3 (Cav3) and other caveolar proteins [43]. The authors observed a progressive loss of Cav3 from caveolae, similar to what is seen after chemical disruption of caveolae via cholesterol depletion, thus indicating a mechanotransductive role of caveolae domains in the heart. A key example of caveolae organizing cellular compartmentation of signalling proteins is the interaction of Cav3 with AC5, PDE4B, and PDE4D [17].

## 5. Microdomains of cAMP Signalling in Healthy Ventricular Myocytes: T-Tubules and Caveolae

Visualization of cAMP signal propagation using flurorescent cAMP sensors has become more sophisticated over recent years, which led to the definition of spatially localized cAMP pools (or nanodomains) [44]. These pools may be located either within a close proximity to receptors and ion channels, or may be distributed throughout the cell. Evidence from adult mouse ventricular myocytes demonstrates the response to stimulation of β_1_-AR is a far-reaching cAMP signal, compared with the local response upon stimulation of β_2_-AR [44]. The difference in the way G_s_-coupled β-ARs compartmentalise cAMP, is linked to two key factors: (i) the localization of the receptor and (ii) the occurrence of cAMP-degrading enzymes. 

To identify the different cAMP pools produced by β-ARs, a combination of SICM and Förster resonance energy transfer (FRET) was utilized. SICM identifies specific microdomains (T-tubule and crest) by producing a detailed topographical image of the cell surface (Figure 1B) [34]. β-AR stimulation was then applied locally to adult rat ventricular myocytes, using the SICM nanopipette to a microdomain of interest. cAMP synthesis was measured, using FRET, in response to local (site-specific) stimulation [16,45,46]. This high-tech approach made it possible for Nikolaev et al. to demonstrate that β_1_-AR-induced cAMP signalling occurs both at the T-tubule and non-T-tubule areas. In contrast the β_2_-AR-induced cAMP response arises from the T-tubules only [16]. In addition, to assessing the cAMP response of β_2_-AR irrespective of its coupling to G_i_-proteins, PTX was used and caused no difference in cAMP compartmentalisation [44]. The larger diffusion pattern of β_1_-AR-cAMP thus explains β_1_-ARs’ ability to produce such a response, as a result of activating far-reaching proteins involved in ECC. 

Caveolae are found in both the T-tubule and the crest membranes in rabbit ventricular myocytes [47]. Caveolae localized at the sarcolemma appear to have a four-fold higher density, compared to those located at the T-tubular membrane [48]. These microdomains act as platforms for the assembly of receptors, signalling components, and their associated targets. They, therefore, undergo dynamic regulation to allow agonist-stimulated receptors to interact efficiently with their respective effectors [49]. Thus the dynamic properties and overall stability of the caveolar microdomains in cardiomyocytes has sparked significant interest over the last decades. Specifically, it is due to these structures being involved in mechanisms responsible for creating distinct β_1_- and β_2_-AR-dependent cAMP signalling. The distribution and clustering of these GPCRs and their downstream signalling components, within or outside of caveolar domains, are important factors in promoting efficient and accurate functional responses to β-ARs stimulation. Multiple studies report the clustering of β_2_-ARs along with its associated signalling elements, including G_s_, G_i_, AC5, and AC6, and protein kinase A (PKA) within the caveolar membrane fractions [50,51]; which have also been shown to co-immunoprecipitate with Cav3 [24,52]. β_1_-AR, on the other hand has been shown to be located in both caveolar and non-caveolar compartments in adult rat ventricular myocytes [51,53]. Thus, a cholesterol/caveolin-3 rich environment is key in building the macromolecular structure, particularly for β_2_-AR. 

The description of nanodomains of cAMP signalling will be incomplete without mentioning A kinase anchoring proteins (AKAPs), scaffolding proteins which constrain PKA to specific subcellular locations in physical proximity to PKA targets. AKAPs, to a large extent, determine a unique phosphorylation pattern downstream of the kinase. In cells undergoing response to a stimulus, the location of some AKAPs can change, thus altering the functional outcome of cAMP signals. However, the detailed look into the function of AKAPs falls beyond the scope of the present review as it is reviewed extensively elsewhere [54,55].

## 6. PDEs and cAMP Compartmentation in Ventricular Myocytes

PDEs are a family of enzymes that degrade cyclic nucleotides [56,57,58]. By acting as functional barriers, they create subcellular cAMP gradients by preventing its free diffusion throughout the cell [59]. They have been divided into 11 families based on their structure, catalytic properties, and their differential affinity for cyclic nuclotides [60,61]. Each family of PDEs has isoforms, which vary according to the following properties: tissue distribution, intracellular localization, and the involvement in cellular signalling. PDE 1–3, 10, and 11 are responsible for degrading both cAMP and cGMP. PDE 4, 7, and 8 are specific for the hydrolysis of cAMP and PDE 5, 6, and 9 are cGMP-specific [62]. It has been shown that blocking all PDEs, using isobutyl-methyl-xanthine (IBMX), after β-AR stimulation leads to changes in physiological response due to the spatial and temporal regulation of cAMP [63,64]. Therefore, this infers that the extensive assortment of PDEs provides routes for cardiomyocytes to modulate cAMP signalling.

The complexity of the various functional abilities of the PDE families is further increased by the concept of cross-talk between cAMP and cGMP pathways. For example, cGMP stimulates PDE2 leading to degradation of both cAMP and cGMP, thus increases in cGMP can alter cAMP hydrolysis. Cyclic GMP can also activate PDE5 and increase the rate of its own degradation [65,66,67,68]. PDE3 has dual specificity with a higher catalytic rate for cAMP, therefore, it can be considered as a cGMP-inhibited cAMP-hydrolysing enzyme [65,68].

Compartmentation of cAMP to specific microdomains at the plasma membrane and in the cytoplasm requires a vast variety of PDEs, and their presence or absence in a particular location is also species-specific. To explore the functionality of receptors and enzymes, utilizing genetically-engineered knock-out/overexpression models has become a powerful methodology in cardiovascular research. Employing these methods has determined the vast impact that PDE3 and PDE4 have in cAMP-hydrolytic activity and calcium handling [69]. In addition, it has been highlighted that isoforms of a single family of PDEs can localize to specific compartments. PDE3 has been specifically shown to associate with T-tubule microdomains and with internally-organized sarcoplasmic reticulum structures [63,70]. Investigating the function of PDEs using knock-out mice and inhibitors has collectively provided evidence suggesting each PDE subgroup contains isoforms that have different microdomain locations and functions. The use of mouse knock-out models and selective PDE3 inhibitors have indicated PDE3A to be responsible for chronotropic and inotropic effects [71], whereas PDE3B serves to acutely protect the heart after biomechanical stress [72]. Specifically, PDE3A1 controls the Phospholamban (PLB) and sarcoplasmic reticulum calcium ATPase2 (SERCA2) activity and the re-uptake of calcium in the SR, leading to an increase in cAMP-dependent calcium transients, without affecting L-type calcium channels (LTCCs) [72]. Therefore, Movesesian suggests a PDE3A1-specific inhibitor could potentially improve contractile performance and provide therapy for heart failure (HF) [73].

Unlike PDE3, PDE4 is shown to localize to the sarcolemma [70] and play an important role in cardiomyocytes [74,75]. Although the expression of PDE4 is species-specific, the relative amount of PDE4 compared to overall PDE activity is conserved between humans and rodents. However, the overall cAMP-hydrolytic activity of PDE4 is estimated to be higher in rodents compared to humans [74]. The inhibition of PDE4 was specifically shown to extensively increase cAMP levels. In addition, the PDE4 activity has presented to be specifically enhanced by β-AR stimulation [44,56]. PDE4 has also been demonstrated to be a predominant isoform that hydrolyses cytoplasmic cAMP produced following β_1_-AR, but not β_2_-AR stimulation [44]. Melsom et al. demonstrated that combined PDE4 and PDE3 inhibition increased the potency of adrenaline on PTX treated β_2_-AR [76]. This suggests that it is possible that PDE4 plays a significant role with β_2_-ARs. Subgroups of PDE4, mainly: PDE4A, PDE4B, and PDE4D are expressed in the heart (see Conti 2017 [77] for an extensive review on PDE4). Previous studies have shown the importance of PDE4D, in particular through its involvement with the PLB/SERCA2A complex [78], the cardiac ryanodine receptors and the calcium release channel complex [79]. In addition, mice lacking PDE4D develop an age-dependant cardiomyopathy and exercise-induced arrhythmias [79]. Moreover, PDE4D appears to control cAMP levels in subcellular compartments, thus having an impact on myocyte contractility [78]. 

In addition, PDE2 was shown to hydrolyse cAMP after β-AR stimulation in adult mouse ventricular myocytes. Here, the inhibition of PDE2 releases a higher level of cAMP than that after PDE3 inhibition [44]. Although not extensively studied, measuring cAMP in ventricular myocytes demonstrated that PDE2 affects the regulation of heart rate. Inhibition of PDE2 caused an increased heart rate, while overexpression reduced the heart rate [80], suggesting that the expression levels do not directly relate to functional integrity, and that perhaps a minor PDE2 fraction could play a larger role in specific microdomains.

## 7. cAMP Compartmentation and PDEs in Atrial Myocytes

In comparison to ventricular myocytes, cAMP compartmentalisation in atrial myocytes remains relatively poorly investigated. The ultrastructural organisation of atrial myocytes differs with ventricular and this suggests differential receptor localization and spatial pools of cAMP leading to unique signaling patterns. So far, to map signalling differences between atrial and ventricular myocytes two major methods have been utilized: electrophysiological methods and expression analysis of β-AR and PDEs. It has now become apparent that in ventricular myocytes caveolae are involved in controlling β-AR signalling [40,43,45,46,48,49,52,53]. Accordingly, an even higher abundance of caveolae in rat atrial myocytes [34] would suggest their important role in the spatial control of cAMP nanodomains in this cell type. Atrial myocytes are thinner and contain less complex TAT networks. Due to their small size, atrial myocytes may not require a complex internal structural architecture. Utilizing methyl-β-cyclodextrin (MβCD)-treated ventricular rat myocytes to disrupt caveolae, β_1_-AR has been found in both caveolae and non-caveolae regions, while β_2_-AR were confined to the caveolae compartment [53]. It has been demonstrated in human atrial myocytes using the whole-cell voltage-clamp that PDE4 reduces cAMP when combined with either PDE3 inhibition or β-AR stimulation [81]. These earlier studies into atrial myocytes warrant the assumption that although β_1_-AR might have a rather similar role to that seen in ventricular myocytes, β2-AR may assume a larger role, due to these receptors being denser, largely because of the increase in caveolar number. To understand the role of PDEs and to establish the principal differences between atrial versus ventricular cardiomyocytes, expression analysis in the mouse right atria (RA) and right ventricle (RV) was performed. PDE2A was shown to be equally expressed across the RA and RV, whereas PDE3B, PDE4B and PDE4D were all expressed at higher levels in the atria [82]. Similar studies have been conducted in rat and human tissue to determine species-specific differences. In contrast to rodent myocytes, human PDE4 is not the predominant subtype regulating cAMP levels. Nevertheless, it was shown that PDE4 inhibition led to the positive inotropic effect of β-AR stimulation, increasing the susceptibility to atrial arrhythmias, and a marked prolongation in cAMP rise [75]. This work is highly suggestive that PDE4 plays a significant role in compartmentation of cAMP. It is possible that PDE4 is more closely associated with caveolae and less with T-tubules, thus explaining why PDE4 has a stronger impact in atrial cells. What would be interesting is to understand how β-AR signalling is propagated in atrial myocytes, and how this impacts the activity of PDEs to compartmentalize cAMP.

## 8. Failing Myocardium Represents Progressive Reduction in the Complexity of Structure and Receptor Function

Studies in rat ventricular myocytes isolated from failing hearts have shown that the progression of disease causes TAT structure to become more disorganized and diminished [45,83]. In comparison to normal cells, the TAT structure in human chronic unloaded HF also presents a decrease in regularity, and for the first time using 3D imaging, demonstrated a sheet-like T-tubule phenotype [84]. In rats, axial elements were increased during early HF, but reduced as HF progressed [45]. These different tubular structures that are present in failing myocytes are not well characterised. In addition, it is not fully understood what causes the reduced density of the TAT structure.

Lyon et al. have demonstrated that surface structures (Z-grooves) in the human myocardium were lost from the cell surface during HF [85]. A similar study conducted in rats measured the Z-groove index, demonstrating loss of these structures in HF [86]. Hence, it is apparent that the relative changes in the TAT system occur in both large and small animals during HF [27,45,84,85,86,87,88].

The continuous structural adaptation, in response to alterations of mechanical load during HF has become a hallmark of cardiac disease. Patients with severe HF, can be fitted with a left ventricular assist device (LVAD), which reduces the mechanical load to the heart in an attempt to regain cardiac function. Studies on myocytes isolated from such failing hearts have shown that implantation of an LVAD could only be beneficial to those hearts that possess a relatively preserved myocyte TAT structure [84]. In comparison, chronic unloading in rat hearts demonstrated a reduction in the density and regularity of the TAT system, in addition to surface changes [86]. Damage to the heart is inhomogeneous, and so it could be said that TAT density loss would occur to a higher extent in certain regions compared to others. This difference in density would initially be identified by the weak contractility of the heart [36].

**Figure 2:**
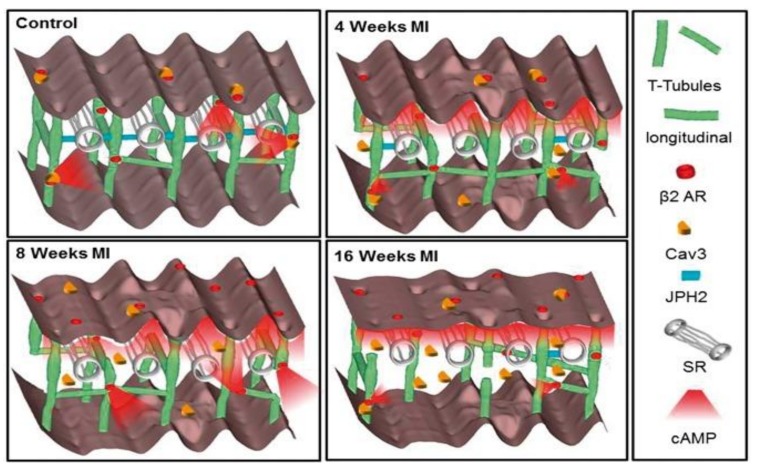
Progressive loss of the t-tubules and the increase of axial (longitudinal) elements of the TAT system demonstrate the change in cAMP signalling in the ventricular myocytes isolated from hearts with myocardial infarction (MI). Focus on the way in which β_2_-ARs are relocated and become more dispersed throughout the cell. As the disease progressively worsens, the loss of TAT structure deviates further and further away, but still attempts to generate signalling that keeps myocytes functional. Taken from Schobesberger et al., 2017 [45].

Structural adaptations that occur in chronic HF come with a functional price—the relocalization of β-ARs. The change in localization of these receptors generates a modified downstream response. Namely, β_2_-AR signalling is altered, such that cAMP signalling is seen to spread from both the crest and T-tubule regions, with smaller signalling amplitudes compared to healthy cells [16]. The alterations seen in β_2_-AR signalling became apparent as early as four weeks post-myocardial infarction (MI) in rats, and progressively worsens over 16 weeks post-MI. It has become evident that as disease progressively worsens, transverse elements are lost and axial elements are gained (Figure 2) [45]. The increase in β_2_-AR cAMP signalling has been thought to be a compensatory mechanism for decreased β_1_-AR cAMP signalling [16]. This indicates that a myocyte attempts to maintain orderly signalling, as conditions deteriorates, which as a result changes the dynamics of cellular signalling.

The expression of Cav3 has also been demonstrated to decrease during HF, and this is important for both the TAT system and caveolae [45,89]. The disruption of Cav3 has been shown to be a significant cause of the far-reaching β_2_-AR-cAMP signals [46]. In support of this, a large portion of β_2_-ARs have been shown to localize to caveolae [24]. Moreover, it was found that caveolar microdomains, as well as housing β-ARs, also contain LTCCs, a critical ion channels activated by cAMP signalling which regulate ECC. A recent study has suggested that the LTCCs located in caveolae have more impact on hypertrophy than contractile function of the heart [90]. Therefore, it is clear that a better understanding is needed of receptors and ion channel function within caveolae and what impact they have on the contractile function of the heart.

As a result of the breakdown of structural microdomains, PDEs are relocated and they lose connectivity with their native environments; thus losing the capacity to perform their inherent function. PDE4 was shown to lose its influence on the confinement of cAMP during early HF [91]. In agreement with this, Lehnart et al. suggested that deficiency in PDE4D was pro-arrhythmogenic and that it was associated with HF in mice [79]. This is suggestive of the PDE4 isoform being localized in the vicinity of cAMP microdomains during healthy cardiac functionally, allowing for precise cAMP compartmentation. Therefore, when structural microdomains are lost, for example in cardiac hypertrophy, not only are β-ARs relocated and functionally altered, but PDE4 that would normally act as a barrier to cAMP diffusion has locally changed [92]. It is believed that the decompartmentation of PDEs initially compensates for the deficient cAMP synthesis by β-ARs, however, the long-term effects of this leads to a loss of cAMP compartments.

PDE2 activity was demonstrated to play a vital role in improving ventricular function. PDE2 is able to provide protective effects under chronic and acute β-AR activation [80]. Whilst the expression of both PDE3 and PDE4 was been shown to be reduced in HF, PDE2 expression was shown to be increased in both animals models and in humans [93]. Therefore, this indicates that cardiac pathophysiology modulates both structure and function of PDEs and the resulting compartmentalization of cAMP. Similar to ventricular cardiomyocytes, the structural architecture of atrial cardiomyocytes is disrupted in disease [70,71,72]. Glukhov et al. reported that T-tubule structure was diminished in failing rat atrial cardiomyocytes, despite cellular hypertrophy. This was accompanied by a deterioration in structural surface topography [34]. In spite of this finding, atrial myocytes have not been studied sufficiently for conclusions to be made on their function during HF. 

Until now, studies on human tissue with persistent atrial fibrillation and HF have shown a decrease in PDE activity, with a selective decrease in PDE4. This has led to the suggestion that PDE4 has a protective role in human atria [75]. It would be interesting to evaluate atrial myocytes structural changes during the HF progression to understand how the TAT structure changes in relation to the caveolae occurrence. This would aid in understanding not only how important Cav3 is to the structure, but how much input caveolae have functionally. Caveolae could be more involved in cellular signalling of atrial versus ventricular myocytes, with resulting changes in PDE compartmentation.

## 9. Biogenesis and Maturation of Structural Microdomains during Differentiation of Myocytes from Stem Cells

There is an increasing need for the development of effective models of cardiac diseases in order to address the progressive decline in structural and physiological “output” of cardiomyocytes. The use of induced pluripotent stem cell-derived cardiomyocytes (iPSC-CMs) provides an unprecedented opportunity to develop in vitro models of numerous cardiac diseases and for drug discovery to drive the field of personalized medicine. Currently, we face major obstacles in generating cardiomyocytes with precise morphological and functional features resembling healthy adult myocytes, making modelling and understanding of treatments for late-onset diseases more challenging. However, the enthusiasm in the field of cardiac regeneration has been passionately fuelled by recent developments in driving iPSC-CM maturation in order to obtain structurally- and functionally-stable cardiomyocytes.

It has been widely shown that cultured and diseased myocytes undergo rapid T-tubular remodelling; such studies highlight the importance of impaired βAR signalling in cardiac disorders. There is now a particular interest in understanding the developmental processes involved in early cardiomyocytes as many studies have shown that myocytes of failing hearts tend to dedifferentiate into foetal-like phenotypes. On the other hand, the activation of dormant pathways developed early in cellular differentiation has also been suggested [92]. Due to the known challenges in obtaining human embryos for such detailed investigations, many studies resort to the use of iPSC-CMs as an in vitro model of cardiomyogenesis. Below, we discuss the development of cardiac microdomains in early cardiomyocytes and the emergence of β-AR-induced cAMP signalling.

T-tubules are thought to be absent in embryonic and neonatal cardiomyocytes [94] and only begin to develop after birth [95]. It is well understood that cultured and diseased cells rapidly undergo a process of T-tubular remodelling; however, very little is known about the processes involved in T-tubule biogenesis. Understanding the underlying mechanisms of T-tubule formation and correct clustering of ion channels within these microdomains would reveal the key processes required for iPSC-CMs to mature into an adult cardiomyocyte phenotype with functionally-developed membrane structures for efficient ECC machinery.

Multiple membrane scaffolding proteins have been associated with T-tubule biogenesis, such as Cav3, junctophilin-2 (Jph2), and bridging integrator-1 (Bin1). Recent work by Fu et al. indicates that Bin1 assists trafficking and clustering of LTCCs, microdomain organization and T-tubule membrane regulation [96]. Furthermore, they showed that transgenic mice with deletions of these individual genes reveal that the primary t-tubule invaginations still exist [96]. This indicates that a single protein alone is not sufficient for tubulogenesis. Rusconi et al. have developed a mimetic peptide (MP) which functions by modulating the trafficking and life cycle of the LTCC. This study has shown that introducing this peptide successfully restores physiological levels of LTCC in the plasma membrane [97]. This may indicate a potential to drive correct channel localization within the cell membrane. Healthy adult cardiomyocytes are characterized by sarcomeric structures that consist of parallel myofilaments, sarcomeric Z-disk, A-, I-, and H-bands which, altogether, allow for an effective and efficient ECC; therefore, maturation of these structures is pivotal for successfully driving maturation of sarcomeres and T-tubules of early cardiomyocytes. Wang et al. have recently shown that long-term treatment with Puerarin, a traditional Chinese medicine used for the treatment of cardiovascular disease, such as MI, can improve myofibril arrays and significantly enhance T-tubule development in embryonic stem cell-derived cardiomyocytes. The mechanism of action has been suggested through the repression of a specific micro-RNA, miR-22, an important target of Cav3, and the up-regulation of Cav3, Bin1, and Jph2 transcript levels [98]. This strongly supports the role of these proteins in T-tubule biogenesis and stability.

While early myocytes do not have well-developed tubular structures, they possess abundant caveolae structures that may represent sites where receptors and their associated signaling molecules are clustered. Furthermore, it has been suggested that caveolae may be precursors of T-tubules in early cardiomyocytes, or at least fulfil some of the same signalosome functions [99]. Cav3 induces the formation of caveolae, which plays an important role as a cell signaling hub and in the developing T-tubule system. In a recent study maturation of caveolae in human iPSC-CMs, it has been found to be dramatically increased at day 60 in culture and further increased by day 90, as reflected by the *Cav3* gene and protein expressions [100]. Furthermore, a study conducted by Rebiero et al. also addressed the issue of the foetal-like misalignment of iPSC-CM membrane structures. They reported that culturing cells on micropatterns helped mature the alignment of myofibrils and improved contractile activity, calcium flow, cell electrophysiology, as well as T-tubule formation [101]; thus, providing cells with specific mechanical cues may encourage cell maturation. 

The positive and negative chronotropic responses to isoproterenol and carbamylcholine demonstrated the presence of functional adrenergic and cholinergic receptors, respectively, in pacemaker cells. A major pathway of the β-AR–dependent chronotropic response is the activation of AC and the consequent rise in cytosolic cAMP and stimulation of PKA. The positive chronotropic effect exerted by forskolin, a direct activator of AC, and by IBMX, a phosphodiesterase inhibitor, suggests that this signalling pathway is already present early in human cardiomyocyte differentiation [102]. Primitive β-AR signalling responses have been detected as early as 13–15 days of differentiation of mouse and human embryonic stem cell-derived cardiomyocytes (ESC-CMs) [92].

Jung et al. further investigated the evolution of the expression and function of the components involved in the βAR signalling pathway in early human iPSC-CMs. It was found that β_2_ARs are the primary source of cAMP/PKA signalling in “early” cardiomyocytes; whereas increasing time in culture leads to the β_1_-AR dependent cAMP production to increase to 60% rise from day 30 to day 60 [99]. Similarly, Wu et al. reported a dramatic increase in β_2_AR gene expression at days 12, 30, and 60 of differentiation, although no significant changes in β_1_AR were seen until day 30. Furthermore, with the use of β_1_AR- and β_2_AR-specific blockers they showed that iPSC-CMs only responded to β_2_-AR receptor activation. At day 60, a switch of β-AR subtype dependence was seen, indicating a dynamic regulation of the receptor dependence of the β-AR signalling pathway during maturation of cardiac myocytes. They also highlighted a significant increase in AC expression after day 12 of differentiation, a level comparable to that of adult human left ventricle tissue. In addition, both PDE3A and PDE4D expression rose by day 60 of maturation [103]. 

The dysregulation of the β-AR pathway is associated with a spectrum of cardiac diseases. For instance, an iPSC model of dilated cardiomyopathy has shown a blunted response to isoproterenol stimulation. Further, epigenetic changes of the PDE genes were found in tissue from a DCM patient and in iPSC-CMs derived from this, leading to an up-regulation of *PDE2A* and *PDE3A* genes [103]. However, these signalling mechanisms have only been minimally investigated, and that too at the whole-cell level. To understand the full functional capacity of these critical regulatory mechanisms in iPSC-CMs, in-depth investigation into how compartmentation can direct these pathways at the subcellular levels remains to be performed.

## 10. Conclusions

Understanding the detailed organization of cAMP signalling microdomains in mature myocytes of both atria and ventricles will provide an excellent reference point to which immature or failing myocytes are to be compared. Progressive changes in the microdomain structure and function in HF and dedifferentiation may be deconstructed in order to attempt to reverse these changes. The success of this reversal may correlate with the success of inducing maturation of hiPSC-derived myocytes. In particular, the role or assembly of nascent T-tubules within, or in the vicinity of, caveolae needs further investigation. Equally, PDE isoforms, which are specific for different microdomains need to be further characterized.
